# Septic Embolic Encephalitis Following Cardiac Valve Replacement

**DOI:** 10.7759/cureus.51628

**Published:** 2024-01-03

**Authors:** Dallin Judd, Jake Oldham, James Lish

**Affiliations:** 1 Medicine, Texas College of Osteopathic Medicine, University of North Texas Health Science Center, Fort Worth, USA; 2 Neuroradiology, Chandler Radiology Associates, Chandler, USA

**Keywords:** disease progression, diagnostic imaging, bioprosthetic valve, enterococcus, cardiac valve replacement, septic embolic encephalitis

## Abstract

This paper presents a detailed case study of a 48-year-old male who underwent ascending aortic aneurysm repair with a bioprosthetic valve five years prior and subsequently developed septic embolic encephalitis, an infrequent yet critical complication following cardiac valve replacement. The patient exhibited an array of initial symptoms, including generalized weakness, fatigue, fevers, chills, diarrhea, and altered mentation. Microbiological analysis of blood cultures revealed the presence of *Enterococcus*, and echocardiogram examination demonstrated vegetation on the prosthetic valve. To assess disease progression, diagnostic imaging, including CT scans and MRIs, was conducted at various time points. The imaging results unveiled several abnormalities, including subarachnoid and parenchymal bleeding, cortical infarcts, cerebritis, and meningitis. Additionally, splenic and renal infarcts were observed through an abdominal CT scan. This case report accentuates the paramount role of diagnostic imaging in corroborating suspected septic embolic encephalitis while underscoring the significance of appropriate management of patients with a history of cardiac valve replacement, thereby emphasizing the urgency of timely intervention.

## Introduction

Septic embolic encephalitis stands as a rare and potentially life-threatening complication that may manifest subsequent to cardiac valve replacement procedures [1]. The risk of prosthetic valve infection significantly exceeds that of native valves, with an approximate incidence of 1% at 12 months and 3% at 60 months following surgery [2]. The migration of infected emboli originating from the prosthetic valve can instigate embolic infarcts in various organs, including the brain. Swift and accurate diagnosis, along with appropriate management, remains pivotal in forestalling further complications and enhancing patient outcomes. In this case report, we furnish a comprehensive analysis of a patient who developed septic embolic encephalitis five years after receiving a bioprosthetic valve during ascending aortic aneurysm repair. We present an overview of the patient's clinical history, physical examination findings, and the results of diagnostic imaging and pathological tests, which sheds light on the diagnostic challenges and therapeutic considerations entailed by this condition.

## Case presentation

A 48-year-old male with a history of ascending aortic aneurysm repair with bioprosthetic aortic valve replacement five years prior presented to the hospital with complaints of generalized weakness, fatigue, fevers, chills, diarrhea, and altered mentation. The patient's symptoms had been progressively worsening over the past two weeks. On physical examination, the patient was febrile and tachycardic. The rest of the cardiovascular and respiratory examinations were unremarkable. Neurological examination revealed mild disorientation and decreased cognitive function. Laboratory investigations showed leukocytosis and an elevated C-reactive protein level. Additional laboratory investigations revealed abnormal blood values, including elevated presepsin levels, prolonged prothrombin time, and partial thromboplastin time, indicative of a systemic inflammatory response and coagulopathy. Blood cultures were positive for a gram-positive cocci, specifically identified as *Enterococcus *and empirical broad-spectrum antibiotics were initiated. Imaging studies, including CT scans and MRIs, were performed to evaluate the extent of the disease.

Transesophageal echocardiography demonstrated the presence of vegetation on the aortic valve with characteristics suggestive of high embolic risk, including mobility and size exceeding the typical threshold. These findings, in conjunction with the patient's neurological symptoms and imaging results, strongly suggested a causal link between valve endocarditis and cerebral embolic events.

The initial MRI scans revealed subarachnoid and parenchymal bleeding, small cortical infarcts, and evidence of cerebritis, along with pachymeningeal and leptomeningeal enhancement. Given the rarity of subarachnoid hemorrhage in such patients, its presence was intriguing. A potential explanation for the subarachnoid hemorrhage might be the combination of infective endocarditis-related embolic events and the patient's altered coagulation status, as indicated by his prolonged prothrombin and partial thromboplastin time.

**Figure 1:**
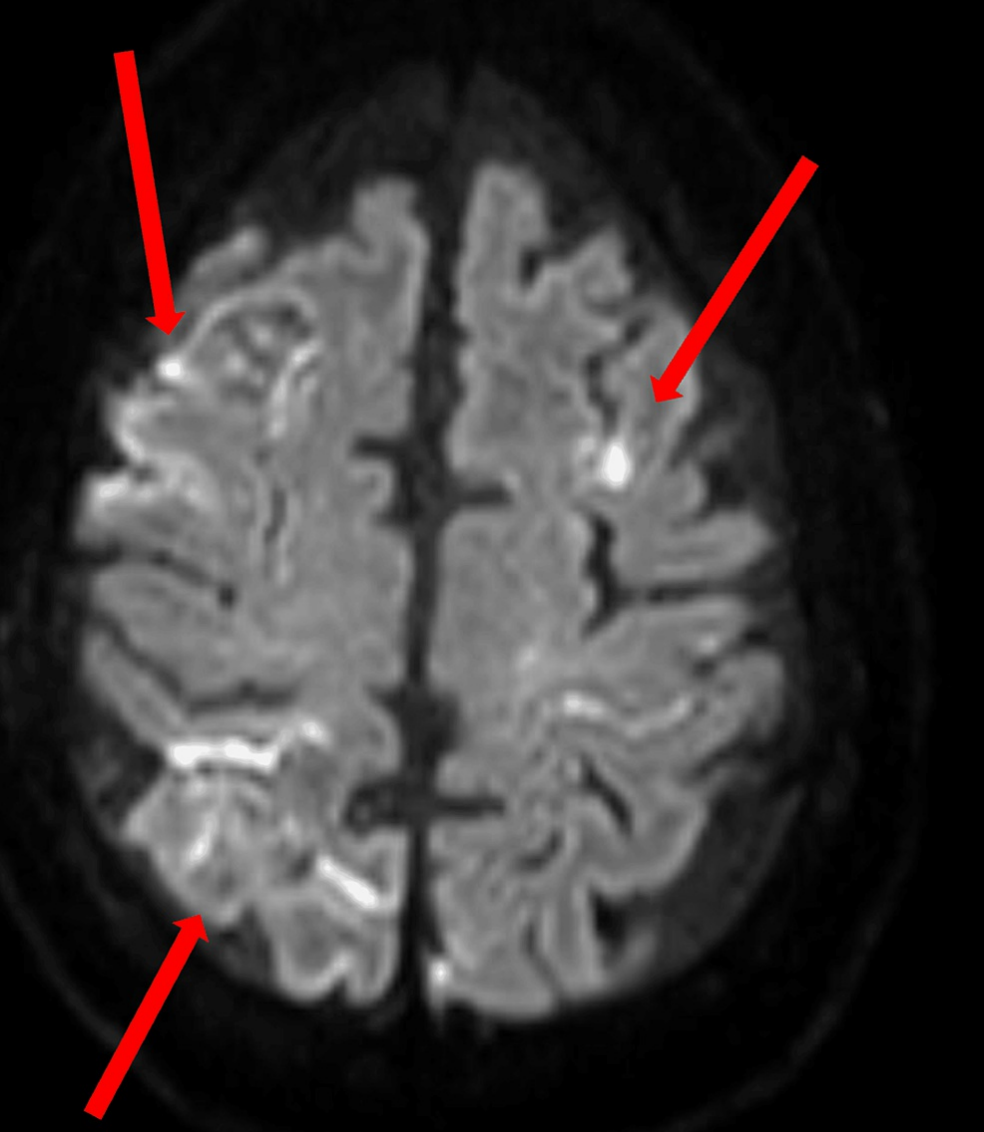
MRI diffusion-weighted imaging shows areas of increased signal, representing areas of acute subarachnoid blood and small cortical infarcts (arrows).

An MRI with diffusion-weighted imaging revealed acute subarachnoid blood and small cortical infarcts (Figure 1).

**Figure 2:**
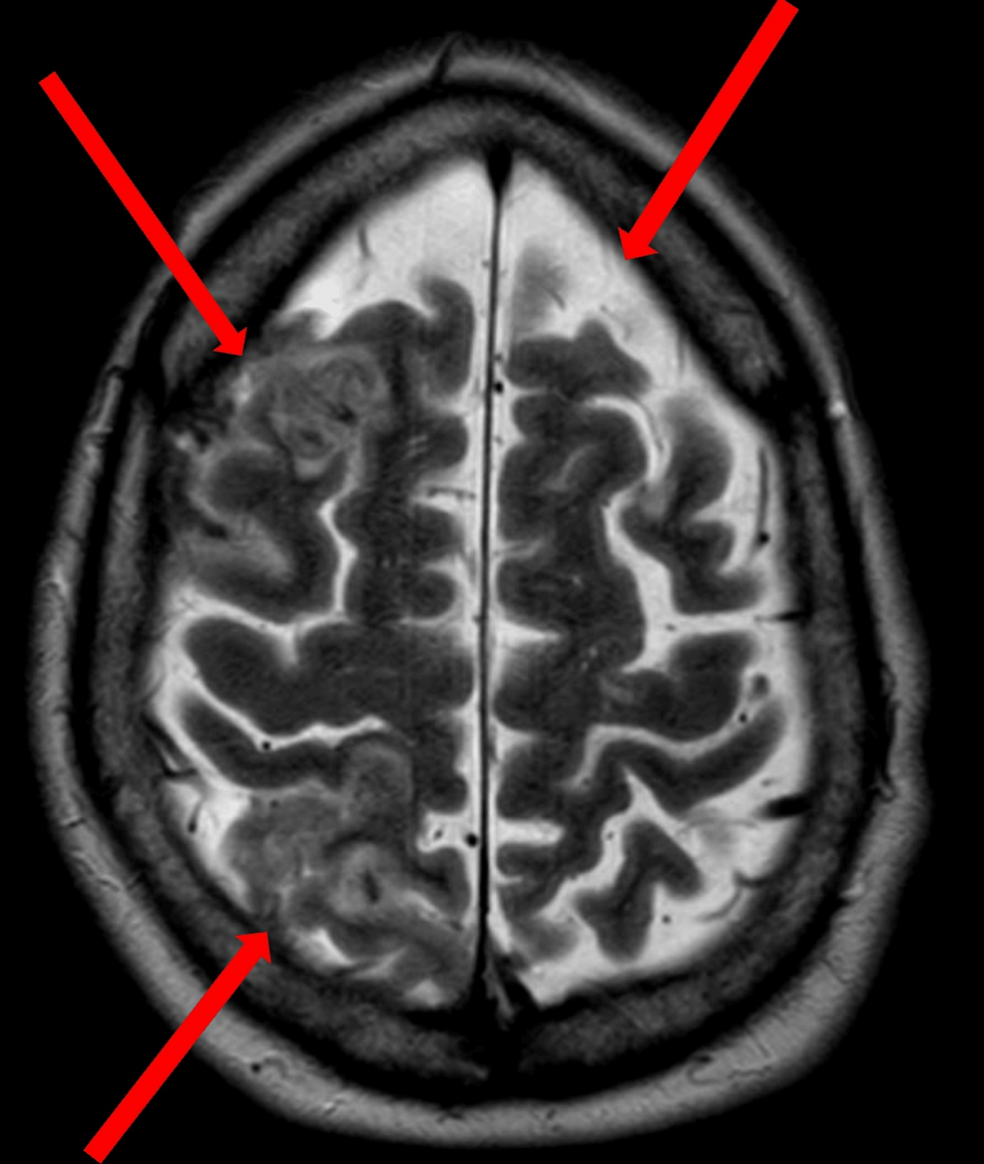
A T2-weighted MRI of the brain without contrast shows areas of increased signal, representing areas of acute subarachnoid blood and small cortical infarcts (arrows).

Similarly, T2-weighted MRI of the brain without contrast also showed acute subarachnoid blood and small cortical infarcts (Figure 2).

**Figure 3:**
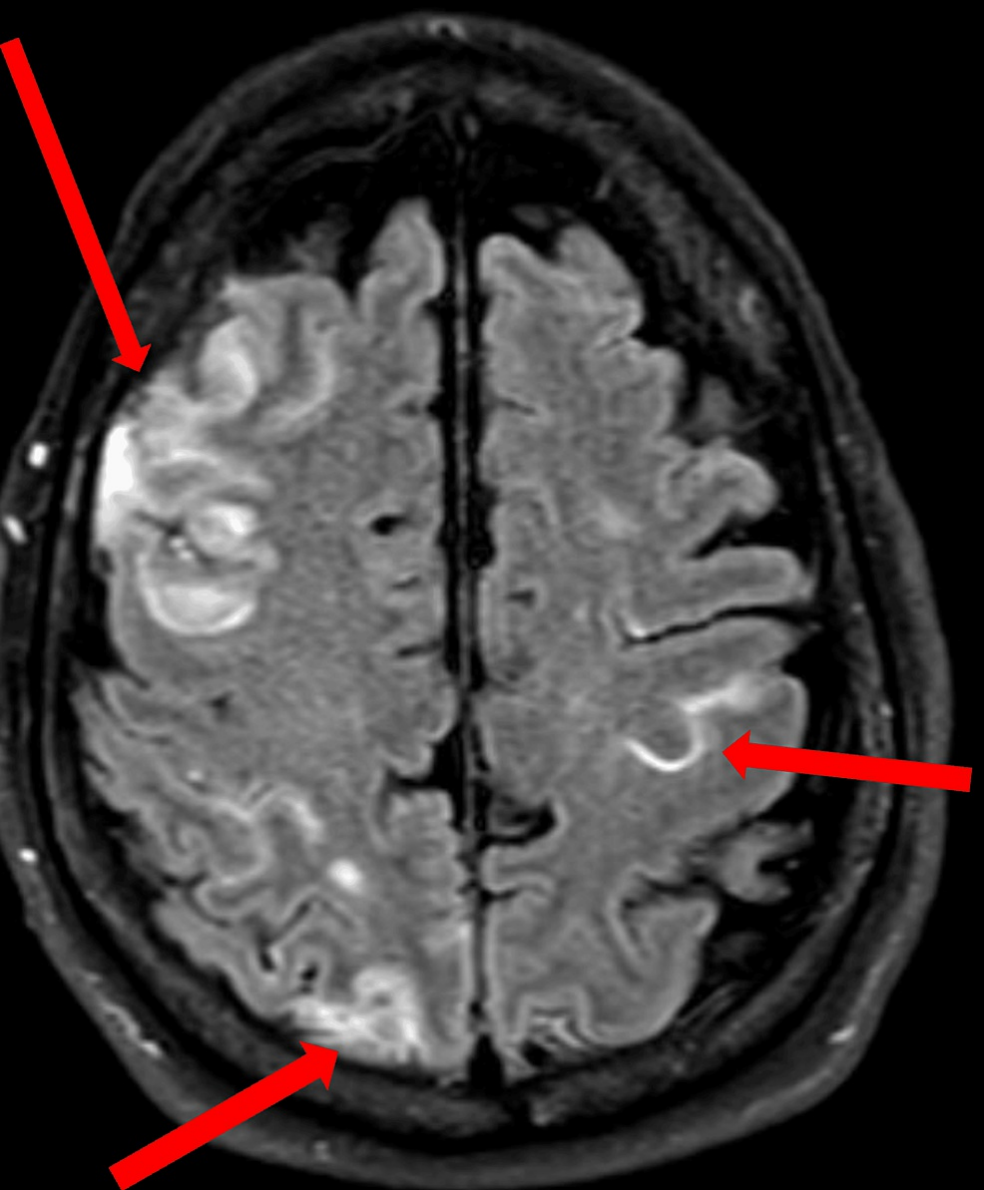
A post-contrast fluid-attenuated inversion recovery (FLAIR) sequence MRI of the brain shows subarachnoid hemorrhage with areas of cortical and leptomeningeal enhancement consistent with cerebritis, subarachnoid hemorrhage, and meningitis (arrows).

A post-contrast fluid-attenuated inversion recovery (FLAIR) sequence MRI of the brain showed findings consistent with cerebritis, subarachnoid hemorrhage, and meningitis (Figure 3).

**Figure 4:**
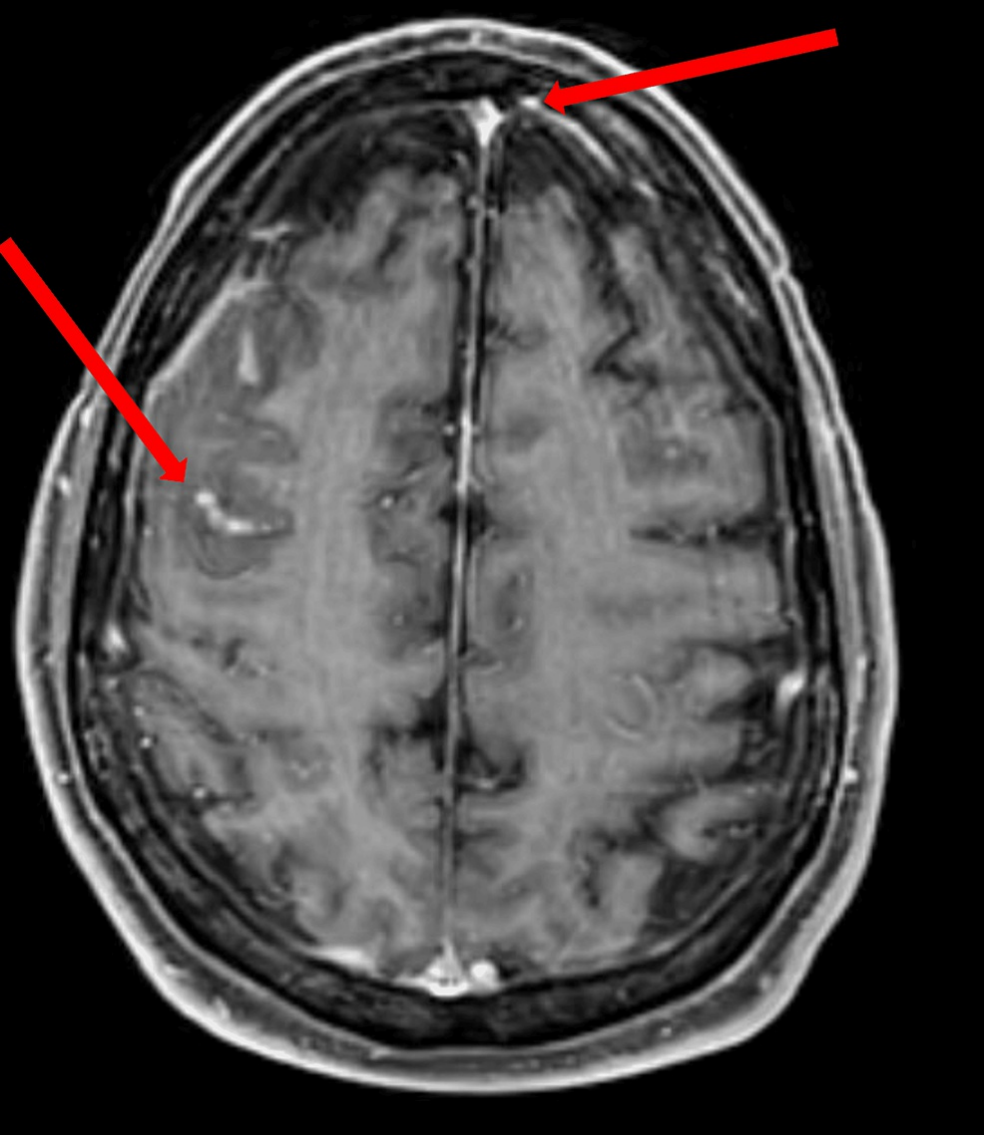
A T1 post-contrast MRI brain demonstrates pachymeningeal leptomeningeal enhancement as well as areas of cortical edema (arrows).

A T1 post-contrast MRI showed pachymeningeal leptomeningeal enhancement and areas of cortical edema (Figure 4).

**Figure 5:**
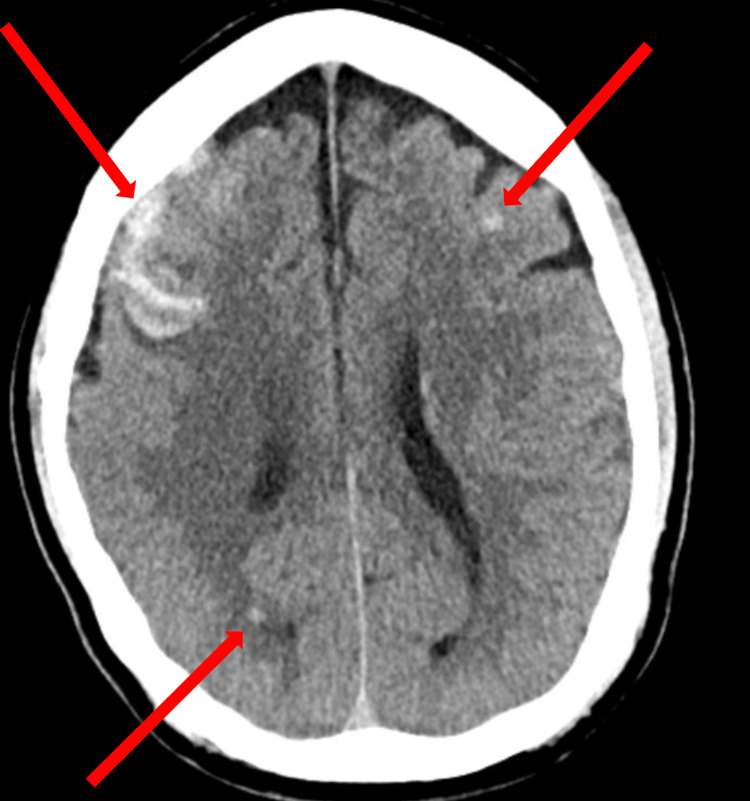
A non-contrast CT scan of the head shows right frontal subarachnoid and subdural hemorrhage with scattered small parenchymal bleeds (arrows).

A non-contrast CT scan of the head showed a right frontal hemorrhage with scattered small parenchymal bleeds (Figure 5).

**Figure 6:**
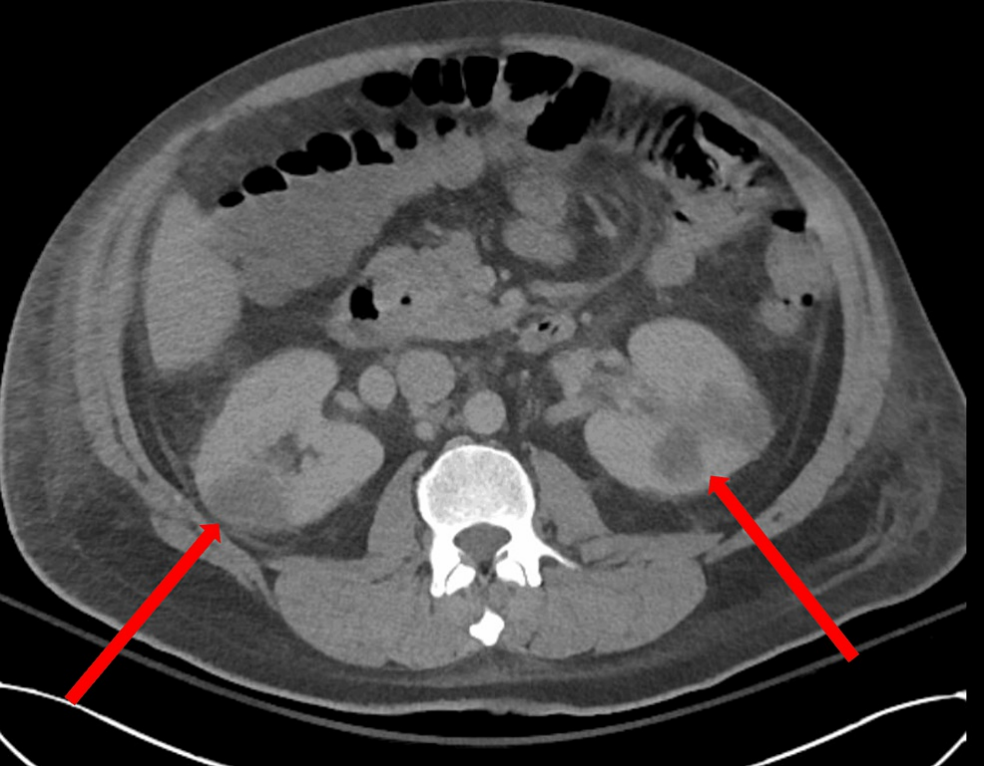
A non-contrast CT scan of the abdomen demonstrates bilateral renal cortical infarcts (arrows).

A non-contrast CT scan of the abdomen showed evidence of renal infarcts (Figure 6).

**Figure 7:**
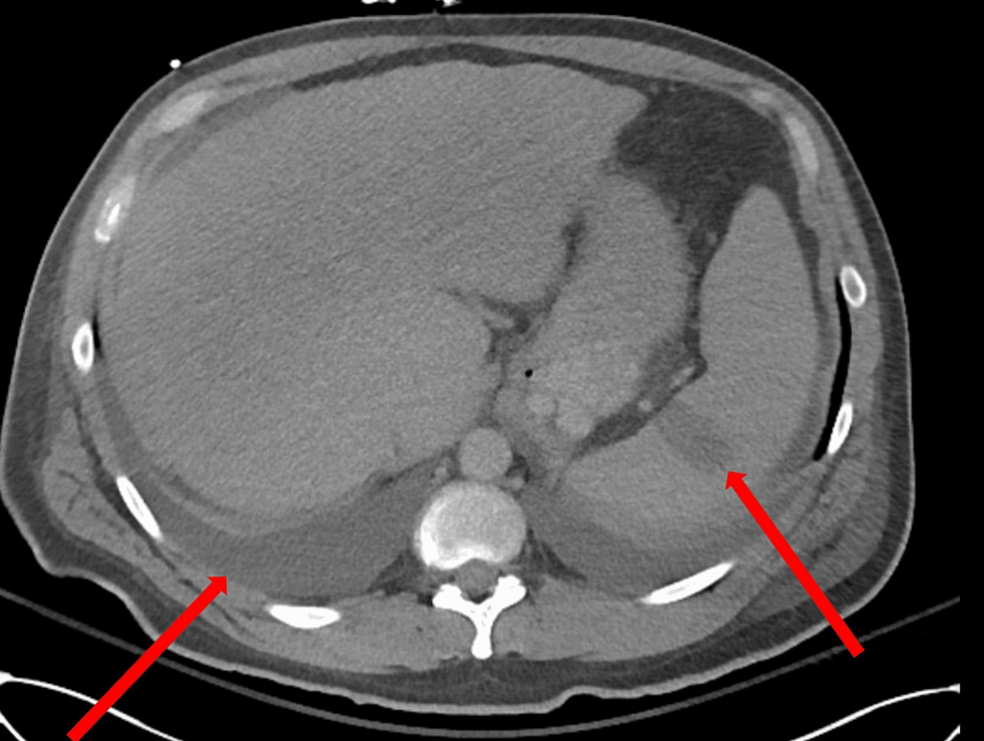
A non-contrast CT scan of the abdomen demonstrates splenic infarct and small pleural effusions (arrows).

Additionally, a non-contrast CT scan of the abdomen demonstrated splenic infarcts and small pleural effusions (Figure 7).

**Figure 8:**
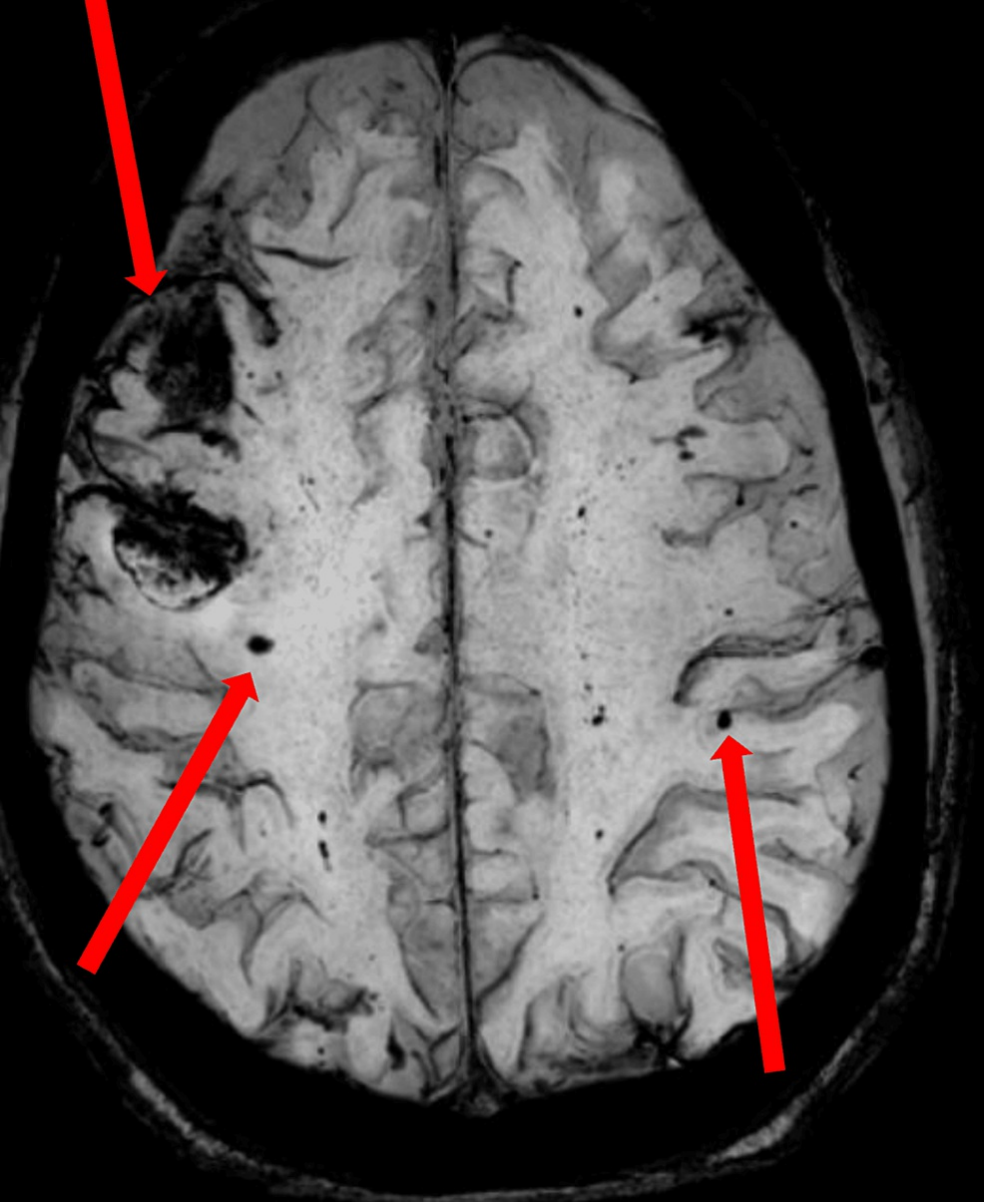
Follow-up MRI susceptibility-weighted imaging shows numerous tiny parenchymal bleeds, infarcts, and extra-axial hemorrhages (arrows).

A second MRI of the brain conducted 15 days later, demonstrated a developing abscess with evolving tiny parenchymal bleeds or infarcts and extra-axial hemorrhage on susceptibility-weighted imaging (Figure 8).

**Figure 9:**
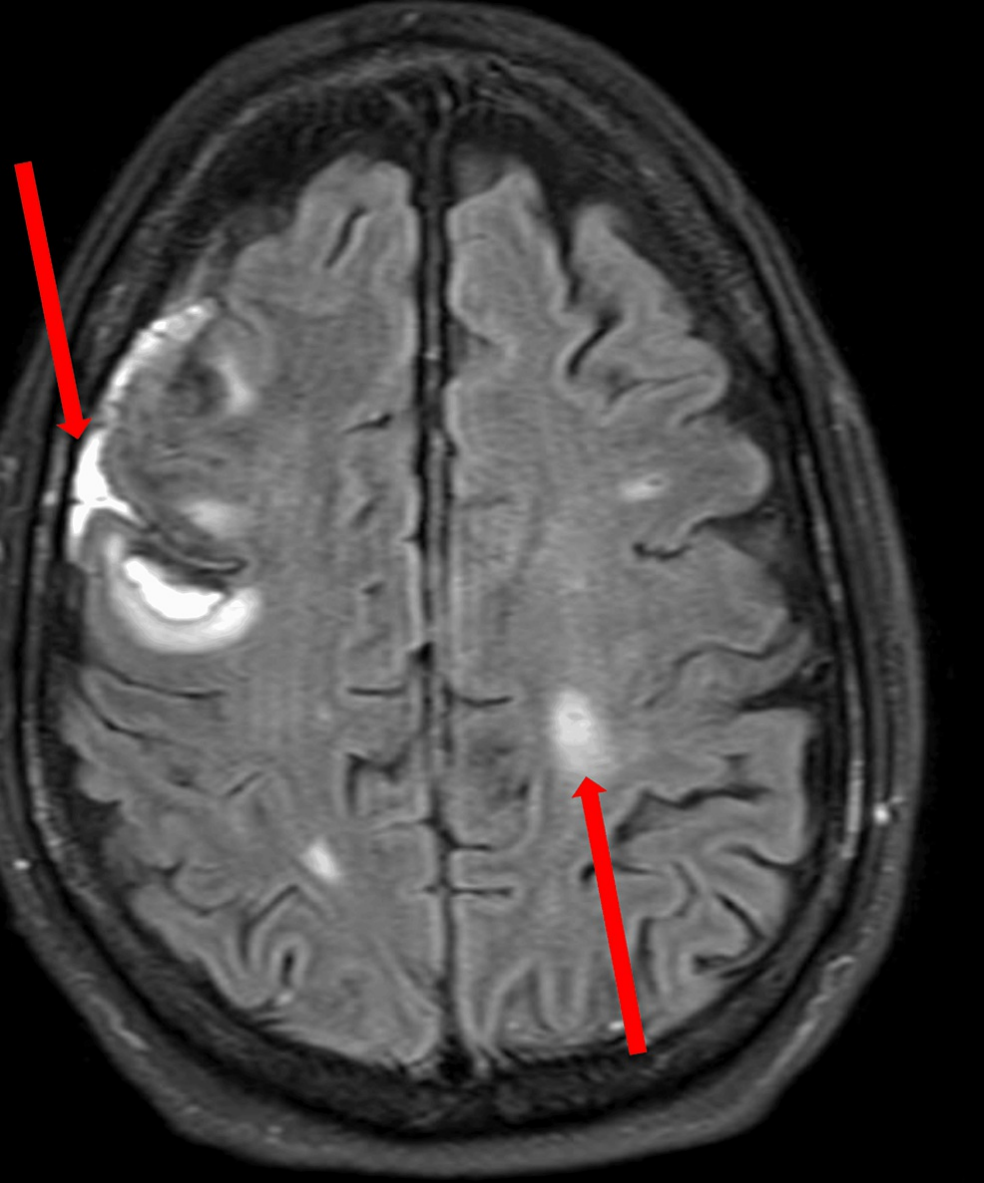
The FLAIR imaging shows evolving extra-axial and parenchymal hemorrhages with areas of cerebritis; phlegmonous changes are visualized on the right (arrows). FLAIR: fluid-attenuated inversion recovery

The FLAIR imaging revealed evolving extra-axial and parenchymal hemorrhage, with areas of cerebritis and phlegmonous changes on the right (Figure 9).

These imaging findings, along with positive blood cultures for *Enterococcus *and the presence of splenic and renal infarcts on CT scans, supported the diagnosis of septic embolic encephalitis following the patient's previous cardiac valve replacement procedure [3]. The patient was diagnosed with septic embolic encephalitis following the previous cardiac valve replacement procedure. Treatment consisted of intravenous antibiotics tailored to the specific pathogen, with a planned duration of six weeks. Additionally, the patient underwent acute rehabilitation to address and manage the neurological sequelae and cognitive deficits associated with the condition.

## Discussion

Infectious endocarditis refers to the inflammation of the heart's inner lining (endocardium) and its valves that separate the four chambers [4]. Notably, around 20%-40% of patients with infectious endocarditis experience neurological complications [5-7]. These complications fall into four categories: hemorrhagic infarct, ischemic infarct, intracranial infection, and mycotic aneurysm [7]. Brain abscesses are rare and occur in less than 1% of all neurological complications [7]. The specific bacteria causing the infection were identified through a blood culture. The presence of *Enterococcus *in this case, typically associated with urinary tract infections, could be linked to the patient's history of cardiac surgery and prosthetic valve placement, which are known risk factors for *Enterococcus *bacteremia, independent of a urinary source. Furthermore, transesophageal echocardiography revealed infection-related growths on the aortic valve, known as aortic valve vegetation. This case report highlights the diagnostic and management challenges encountered in a patient with a history of ascending aortic aneurysm repair and bioprosthetic valve placement.

The patient presented with constitutional symptoms, altered mentation, and positive blood cultures indicating systemic infection [4]. Diagnostic imaging played a crucial role in assessing the extent of embolic involvement and guiding treatment decisions. The initial MRI revealed subarachnoid and parenchymal bleeding, cortical infarcts, cerebritis, and meningeal enhancement, consistent with septic embolic encephalitis. The follow-up MRI showed evolving findings, including progressive meningitis and parenchymal hemorrhage, highlighting the dynamic nature of the disease and the need for serial imaging to monitor its progression.

The positive blood cultures confirmed the presence of a pathogenic organism, underscoring the significance of obtaining cultures in suspected cases. Identifying the specific bacterial pathogen as gram-positive cocci, particularly *Enterococcus*, in the blood cultures enabled tailored antibiotic treatment. Since *Enterococcus *is known for its resistance to many antibiotics, a targeted approach was necessary [8]. The choice of antibiotics was guided by the susceptibility profile of the isolated strain. Usually, intravenous antibiotics in combination are initiated to ensure broad coverage and address the possibility of polymicrobial infection [9]. *Enterococcus *is often susceptible to ampicillin and gentamicin, and these agents are frequently combined for synergistic activity [10]. In some instances, vancomycin may be considered as an alternative if resistance is suspected or confirmed [10]. Antibiotic therapy is generally prolonged, lasting at least six weeks to ensure complete eradication of the infection and prevent relapse [11].

Besides antibiotic treatment, managing the neurological sequelae and cognitive deficits associated with septic embolic encephalitis requires supportive care and close monitoring. In this case, the patient underwent acute rehabilitation to address these issues. While the main focus of infective endocarditis leading to septic embolic encephalitis is on the brain, the potential involvement of other organs should not be overlooked. Splenic and renal infarcts are common and noteworthy complications of infectious endocarditis [12, 13]. The occurrence of these infarcts suggests the spread of infected emboli through the bloodstream and can significantly impact patient management. Splenic and renal infarcts occur when infected emboli obstruct the blood vessels supplying these organs, leading to tissue death. The compromised function of the spleen due to infarction can weaken the immune system's response to the infection. Similarly, renal infarcts can impair kidney function, affecting waste filtration and fluid-electrolyte balance. The presence of splenic and renal infarcts underscores the systemic involvement and seriousness of the condition. Vigilant monitoring, appropriate management of complications, and prompt treatment are essential to mitigate potential long-term consequences and improve the overall prognosis for individuals with septic embolic encephalitis.

Effectively managing septic embolic encephalitis requires a multidisciplinary approach involving infectious disease specialists, neurologists, neurosurgeons, and cardiac surgeons [14]. Early recognition, appropriate antibiotic therapy tailored to the identified pathogen, and timely surgical intervention if needed are vital for improving outcomes and reducing morbidity and mortality. This case report underscores the importance of clinical suspicion, appropriate diagnostic imaging, and multidisciplinary collaboration in managing septic embolic encephalitis after cardiac valve replacement.

Further research is necessary to better understand the optimal duration and combination of antibiotics for treating enterococcal septic embolic encephalitis and to explore strategies for preventing embolic complications in patients with prosthetic valves [15]. Continued efforts in research and clinical practice will contribute to improved diagnostic strategies and treatment approaches, ultimately leading to better outcomes for patients affected by this challenging condition.

## Conclusions

This case report underscores the diagnostic challenges and therapeutic considerations involved in managing septic embolic encephalitis following cardiac valve replacement. The presented case highlights the significance of prompt recognition of constitutional symptoms, altered mentation, and positive blood cultures, which led to the diagnosis of septic embolic encephalitis. Diagnostic imaging, including MRI, played a crucial role in assessing the extent of embolic involvement and monitoring disease progression. The successful management of septic embolic encephalitis necessitates a multidisciplinary approach involving infectious disease specialists, neurologists, neurosurgeons, and cardiac surgeons. Treatment typically involves a prolonged course of tailored intravenous antibiotics, accompanied by acute rehabilitation to address associated neurological sequelae. By emphasizing the importance of clinical suspicion, appropriate diagnostic imaging, and collaborative management, this case report adds to the existing literature on septic embolic encephalitis and provides valuable insights for clinicians involved in the care of patients with a history of cardiac valve replacement. This case underscores for clinical physicians the critical importance of early diagnostic measures and multidisciplinary management in improving outcomes for patients with septic embolic encephalitis post-cardiac valve replacement. Continued research in this field is essential to further improve diagnostic strategies and optimize treatment outcomes.
